# Three-Dimensional Observations of an Aperiodic Oscillatory Gliding Behavior in Myxococcus xanthus Using Confocal Interference Reflection Microscopy

**DOI:** 10.1128/mSphere.00846-19

**Published:** 2020-01-29

**Authors:** Liam M. Rooney, Lisa S. Kölln, Ross Scrimgeour, William B. Amos, Paul A. Hoskisson, Gail McConnell

**Affiliations:** a Strathclyde Institute of Pharmacy and Biomedical Sciences, University of Strathclyde, Glasgow, United Kingdom; b Department of Physics, SUPA, University of Strathclyde, Glasgow, United Kingdom; University of Wisconsin—Madison

**Keywords:** 3D imaging, bacterial motility, gliding motility, label free, live-cell imaging, optical microscopy

## Abstract

The deltaproteobacterium Myxococcus xanthus is a model for bacterial motility and has provided unprecedented insights into bacterial swarming behaviors. Fluorescence microscopy techniques have been invaluable in defining the mechanisms that are involved in gliding motility, but these have almost entirely been limited to two-dimensional (2D) studies, and there is currently no understanding of gliding motility in a three-dimensional (3D) context. We present here the first use of confocal interference reflection microscopy (IRM) to study gliding bacteria, revealing aperiodic oscillatory behavior with changes in the position of the basal membrane relative to the substrate on the order of 90 nm *in vitro*. First, we use a model planoconvex lens specimen to show how topological information can be obtained from the wavelength-dependent interference pattern in IRM. We then use IRM to observe gliding M. xanthus bacteria and show that cells undergo previously unobserved changes in their adhesion profile as they glide. We compare the wild type with mutants that have reduced motility, which also exhibit the same changes in the adhesion profile during gliding. We find that the general gliding behavior is independent of the proton motive force-generating complex AglRQS and suggest that the novel behavior that we present here may be a result of recoil and force transmission along the length of the cell body following firing of the type IV pili.

**IMPORTANCE** 3D imaging of live bacteria with optical microscopy techniques is a challenge due to the small size of bacterial cells, meaning that previous studies have been limited to observing motility behavior in 2D. We introduce the application of confocal multiwavelength interference reflection microscopy to bacteria, which enables visualization of 3D motility behaviors in a single 2D image. Using the model organism Myxococcus xanthus, we identified novel motility behaviors that are not explained by current motility models, where gliding bacteria exhibit aperiodic changes in their adhesion to an underlying solid surface. We concluded that the 3D behavior was not linked to canonical motility mechanisms and that IRM could be applied to study a range of microbiological specimens with minimal adaptation to a commercial microscope.

## Abstract (precis)

3D imaging of live bacteria with optical microscopy techniques is a challenge due to the small size of bacterial cells, meaning that previous studies have been limited to observing motility behavior in 2D. We introduce the application of confocal multiwavelength interference reflection microscopy to bacteria, which enables visualization of 3D motility behaviors in a single 2D image. Using the model organism Myxococcus xanthus, we identified novel motility behaviors that are not explained by current motility models, where gliding bacteria exhibit aperiodic changes in their adhesion to an underlying solid surface. We concluded that the 3D behavior was not linked to canonical motility mechanisms and that IRM could be applied to study a range of microbiological specimens with minimal adaptation to a commercial microscope.

## INTRODUCTION

Bacteria use a number of mechanisms to move through their local environment in response to chemotactic signals, to form communities or to invade their host. The most-studied mode of bacterial motility is flagellar-mediated movement. Other modes, such as the twitching motility displayed by Pseudomonas aeruginosa, use type IV pili (T4P) to direct movement based on the extension, adhesion, and retraction of polar filaments from the leading pole of the cell (1, 2). However, not all bacteria rely solely on extracellular appendages for motility. The phenomenon of gliding motility has been identified in a diverse range of bacterial species spanning various phyla (3–8). The deltaproteobacterium Myxococcus xanthus displays two different modes of gliding motility, adventurous motility and social motility, to seek out nutrients or prey as part of its complex life cycle (4, 9–15).

There are contrasting models proposed to explain the mechanisms underpinning gliding motility (9, 16–19). The focal adhesion complex (FAC) model proposes that FACs form on the basal surface of the cell and attach to the underlying substrate while coupling to the helical MreB cytoskeleton on the cell’s inner membrane (16, 17, 20, 21). It has been shown that FACs translocate linearly from the leading pole as the cell moves forward, which is driven by the force generated by the AglRQS gliding complex, which is associated with the FAC (16, 22). The FAC model requires the basal layer of the cell to be firmly attached to the underlying substrate; however, it remains unclear how the complex is able to traverse the peptidoglycan cell wall without compromising the structural integrity of the cell (8, 19). A second model has been suggested where proton motive force (PMF) generated by AglRQS results in a helical rotation of the MreB cytoskeleton in gliding cells that are firmly adhered to a solid substrate (9, 22–25). In the helical rotation model, stationary foci of fluorescently tagged motor complex subunits have been explained as being a buildup of multiple complexes arrayed in “traffic jams,” which result from areas of differing resistance in the underlying substrate (9, 24). Both models converge where the gliding cell is adhered firmly to the surface of the underlying substrate to facilitate gliding. However, our observations show that cells are not in fact firmly adhered during gliding motility but instead exhibit aperiodic fluctuations in surface adhesion as they glide.

Bacterial gliding motility has mainly been studied using phase-contrast and fluorescence microscopy techniques, which do not provide three-dimensional (3D) information about cell movement (9, 17, 22, 26). We hypothesized that axial changes in cell shape during gliding motility may occur due to the complex nature of underlying mechanisms such as FAC translocation and bulk movement of the cytoskeleton. We reasoned that novel motility behaviors could be visualized using the label-free microscopy technique interference reflection microscopy (IRM) to detect cell shape changes in 3D. This relatively low-cost and easily implemented technique for existing microscope systems has previously been used to study focal adhesion sites of eukaryotic cells on glass substrates (27–34) and microtubule dynamics (35–37). Previous studies have used wide-field IRM to observe gliding motility in *Cytophaga* spp., where rotation and adhesion to glass surfaces were characterized (4). Wide-field IRM has also been used to investigate twitching motility in Pseudomonas fluorescens, where the attachment profile of twitching cells was found to be dependent on the presence of different electrolytes (38). However, wide-field IRM images have a low contrast between the orders of interference due to the short coherence length of the light source. The contrast of higher-order interference fringes can be improved by using IRM in confocal mode where coherent laser light is used and the out-of-focus signal is significantly reduced by incorporating a pinhole before the photodetector (39).

In IRM, the specimen is illuminated via the lens objective, and the same objective serves to capture the signal originating from the interference of reflected light at refractive index boundaries within a live-cell specimen that is plated on a glass coverslip. Methods for adjusting the epicondenser were described previously by Izzard and Lochner and by Verschueren, who recommended that contributions to the image from the dorsal side of the cell (i.e., the side most distant from the cover glass) could be reduced in wide-field mode by opening the illumination aperture fully and that glare could be lessened by reducing a field aperture to illuminate only the cell under observation (40, 41). Confocal scanning optical microscopes introduced in the 1980s were mainly used for epifluorescence microscopy but also proved to work well for IRM when a beam splitter was substituted for the chromatic reflector in the beam path. Indeed, it became possible to study individual microtubules gliding over glass substrates in a motility assay, which had not been reported with wide-field IRM, indicating that the use of confocal optics increases the sensitivity of IRM, perhaps by orders of magnitude (35, 39, 42).

In either wide-field or confocal mode, the IRM image is formed by the same principle. Reflected light originating from the coverslip-medium and the medium-cell interfaces results in constructive and deconstructive interference, dependent on the optical path length difference between both. The resulting fringe pattern can be used to estimate the axial position of the cell surface (see “Background theory,” below) (41, 43). Using IRM, a 5-fold axial resolution enhancement is achieved over conventional wide-field or point-scanning microscopy techniques, where 3D information can be extracted from a two-dimensional (2D) image, thus overcoming the limitations in optical sectioning of thin specimens (i.e., bacteria) (27, 41, 43). An additional benefit of IRM in comparison to other similar methods is that it requires almost no adaptation to a standard confocal or wide-field microscope and is compatible with existing lens objectives. Additionally, no fluorescence labeling of the specimen is required.

The application of IRM to biological specimens has been documented since the 1960s, but the interpretation of IRM data can be difficult (27). Theoretically, the axial resolution of IRM can be as high as 15 nm (27), but in practice, factors such as dense protein aggregates, the transport of dry mass to the basal membrane, changes in local membrane density, and the proximity of intracellular structures to the membrane affect the brightness of the detected IRM image (41, 44). However, in thin specimens such as bacterial cells, where internal shifting of dry mass is unlikely due to the lack of intracellular vesicular transport, IRM remains a viable height-measuring technique (45). Godwin et al. found that separation between the cell and the coverslip on the order of 100 nm can be easily distinguished without an influence from ambiguities introduced by the above-listed factors (4). Others have disputed the ability of IRM to accurately measure close-contact sites in live cells, for example, by imaging the displacement of a thin layer of fluorescent dye between adherent cells and the cover glass with total internal reflection fluorescence (TIRF) microscopy (46). In the present work, it is assumed that the IRM contrast of bacterial specimens is an indication of the height of the cell surface above the substrate (see “Background theory,” below).

This study is the first application of confocal IRM to bacterial specimens. However, previous studies have used similar techniques as a means of contrast enhancement. One such study used reflection interference contrast microscopy (RICM), where polarizing filters and an antiflex objective are used to filter out reflected light outside the specimen plane and increase the image contrast (39, 47, 48), to image only M. xanthus surface detachment from the cover glass (17). TIRF microscopy has also shown that FACs that attach to and rotate the MreB cytoskeleton are found in distinct foci on the basal side of the cell (9, 16). These membrane-associated complexes have been suggested to change the surface topology of the gliding cell depending on the cargo load of the molecular motor (9). More recently, interferometric scattering microscopy (iSCAT), which detects both reflected and scattered light, has been used to observe T4P-mediated twitching motility in P. aeruginosa. In that work, the authors generated 3D illustrations that revealed the role of T4P machinery subunits in extension, attachment, and retraction based on the interference pattern in iSCAT images (49).

## 

### Background theory.

For the image formation theory in IRM, a simplified three-layer model system is assumed, which consists of only the cover glass (*g*), the cell medium (*m*), and the cell (*c*). In this model, the cell medium can be viewed as a thin film with varying height, dependent on how closely the cell is attached to the coverslip. The intensity of the reflected light, *I_g-m_*, at an interface, for example, the cover glass-medium interface (*g-m*), follows the Fresnel equations:

$$I_{g\text{-}m}=I_{0}{\left(\frac{n_{g}-n_{m}}{n_{g}+n_{m}}\right)}^{2}$$

where *I*_0_ is the intensity of the incident light beam and *n_g_* and *n_m_* are the respective refractive indices of the adjacent materials.

The reflected light beams at the cover glass-medium (*g-m*) and the medium-cell (*m*-*c*) interfaces coincide, leading to (de)constructive interference dependent on the optical path length difference, *z*, between the two beams (see Fig. S1 in the supplemental material). The intensity follows *I*(*z*):

$$I\left(z\right)=I_{g\text{-}m}+I_{m\text{-}c}+2\sqrt{I_{g\text{-}m}I_{m\text{-}c}}\cos\left(\frac{4\text{π}n_{m}}{\text{λ}}z+\text{δ}\right)$$

where *n_m_* is the refractive index of the cell medium, λ is the wavelength of the light, and δ is the phase difference (50). Since the refractive index of the cell medium is smaller than the refractive index of the cell, a phase shift occurs upon reflection so that δ equals π (41). Accordingly, deconstructive interference occurs at optical path length differences of *z* = *N*(λ/2*n_m_*), and constructive interference occurs at *z* = (*N* + 1/2)(λ/2*n_m_*), with *N *= 0, 1, 2, … as the interference order. This wavelength dependence can be used to extract the cell topology from IRM images since the overlap of the interference fringes of different wavelengths decreases with increasing distance between the cell and the cover glass. The decreasing fringe overlap increasing the separation between the cell and cover glass, as observable in color-merged IRM images obtained for different wavelengths, results in a clear color ordering along the cell body when the cell is lifted up from the cover glass.

10.1128/mSphere.00846-19.1 FIG S1 Schematic of the IRM model. The model system consists of the cover glass, the cell medium, and the cell body. Reflections occur at both the cover glass-to-medium and the medium-to-cell interfaces. Propagation of the direction of the incident and reflected light is perpendicular to the cover glass. The two reflected light beams interfere (de)constructively, dependent on the optical path length difference. Download FIG S1, TIF file, 1.1 MB. Copyright © 2020 Rooney et al. 2020 Rooney et al. This content is distributed under the terms of the Creative Commons Attribution 4.0 International license.

Common assumptions of this IRM model are that no other refractive index boundary exists in the cell specimen (i.e., that the refractive index of the cell is constant) and that incident and reflected light are perpendicular to the cover glass (28). Also, the impact of the numerical aperture (NA) is neglected, which affects the depth of the field that is imaged. In IRM, high-NA objectives are used to limit the detection of reflection signals to those originating from interfaces close to the cover glass, which establishes an experimental condition that is close to the three-layer system consisting of the cover glass, the cell medium, and the cell body (39).

## RESULTS

### Characterization of the model specimen.

**FIG 1:**
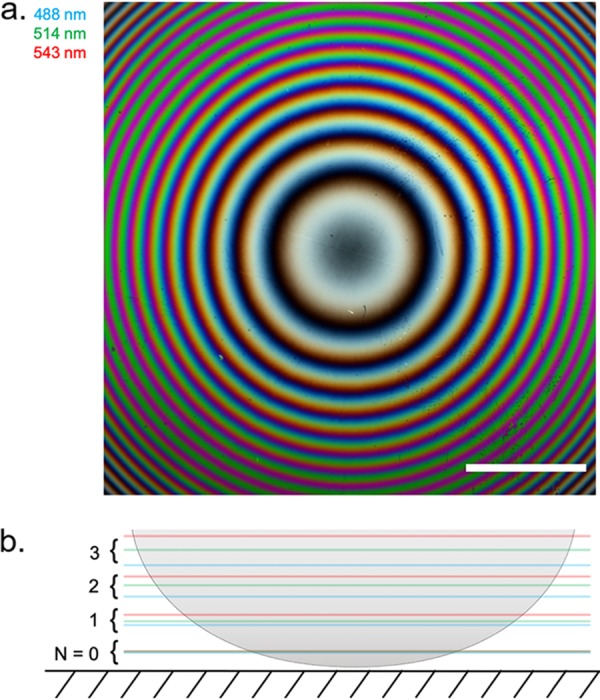
IRM image and schematic diagram of a planoconvex lens specimen. (a) Composite IRM image acquired using wavelengths at 488 nm, 514 nm, and 543 nm, which are false colored as indicated. As the concentric fringes propagate away from the cover glass, we observed spectral separation of the fringes. Bar = 200 μm. (b) Cross-sectional schematic of the lens specimen showing the color ordering of each acquisition wavelength as the fringes propagate axially from the cover glass.

**FIG 2:**
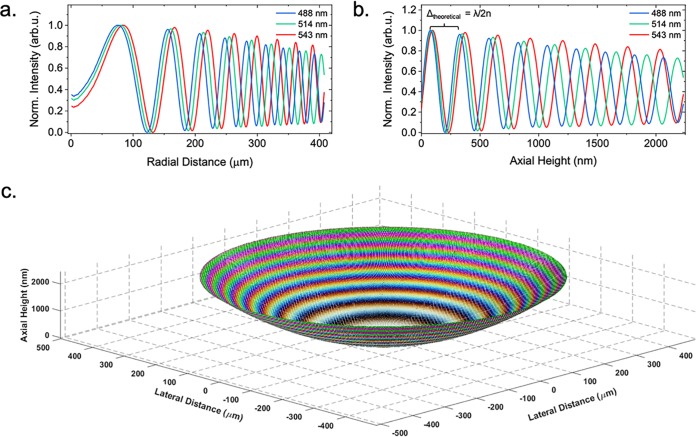
3D reconstruction of a planoconvex lens specimen. (a) Radial intensity profile of the interference fringe pattern shown in Fig. 1a. We observed the fringe periodicity decrease as observed in the RGB IRM image. (b) Using *a priori* knowledge of the lens specimen, the axial height was calculated and used to plot the intensity of each pixel as a function of height. arb.u., arbitrary units. (c) 3D reconstruction of the lens specimen using the known *x*, *y*, and *z* values and intensity extracted from the 2D IRM image.

We characterized the axial height intensity profile of a specimen of known structure to compare with IRM theory by acquiring IRM images at different wavelengths of a planoconvex lens (focal length = 72 mm) that was placed on a cover glass. A composite of IRM images of the lens specimen acquired at 488 nm, 514 nm, and 543 nm is shown in Fig. 1a. In Fig. 1b, a cross-sectional schematic of a planoconvex lens specimen is shown, outlining the axial position of the intensity maxima that are caused by constructive interference for different orders and wavelengths. We analyzed the intensity of the interference fringes by comparing the radial intensity profile with the theory of IRM regarding fringe separation (27). We calculated that the theoretical spacings (λ/2*n*) between the intensity maxima caused by constructive interference for the different wavelengths are 244 nm, 258 nm, and 272 nm (*n *= 1). With the experimental data, we obtained slightly different spacings for the intensity maxima, 249 ± 1 nm, 262 ± 1 nm, and 277 ± 1 nm (Fig. 2b). Thus, experimental and expected values deviated by 2.07%, 1.63%, and 1.91% for the different wavelengths. Additionally, the overlap of the intensity maxima of different acquisition wavelengths decreased with lens-to-coverslip distance (Fig. 2). These observations provided a sense of directionality regarding specimen topology, where the curvature of the planoconvex lens specimen was clear from the acquired images and allowed 3D reconstruction of the lens specimen (Fig. 2c). This means that the assumptions outlined above (see “Background theory”) are appropriate to reconstruct the morphology of simple model systems. Here, a multiwavelength IRM approach provides important additional morphological information over single-wavelength IRM.

### Axial changes along the cell body during gliding motility are independent of AglQ.

To demonstrate the benefit of using confocal IRM over wide-field IRM, we first imaged wild-type M. xanthus on a commercial wide-field system. We demonstrated that low-contrast images are generated in wide-field IRM where interference fringes along the cell cannot be resolved clearly. The raw wide-field data are presented with a magnified region showing gliding cells (Fig. S2a) accompanied by the background-corrected data (Fig. S2b). Interference fringes cannot be clearly resolved in either the raw or corrected data, meaning that a wide-field approach is not suitable for studying the changing adhesion profile of gliding bacterial cells.

10.1128/mSphere.00846-19.2 FIG S2 Wide-field IRM does not provide sufficient contrast to reveal subdiffraction-limited changes in the adhesion profile of gliding bacteria. Shown are unprocessed (a) and background-corrected (*k* = 30) (b) multiwavelength wide-field IRM composite images of M. xanthus (cyan, 450 nm; magenta, 550 nm). A magnified ROI of gliding cells shows low contrast between fringe orders when images are acquired using a wide-field modality. Bars = 20 μm (full field) and 5 μm (ROI). Download FIG S2, TIF file, 2.6 MB. Copyright © 2020 Rooney et al. 2020 Rooney et al. This content is distributed under the terms of the Creative Commons Attribution 4.0 International license.

**FIG 3:**
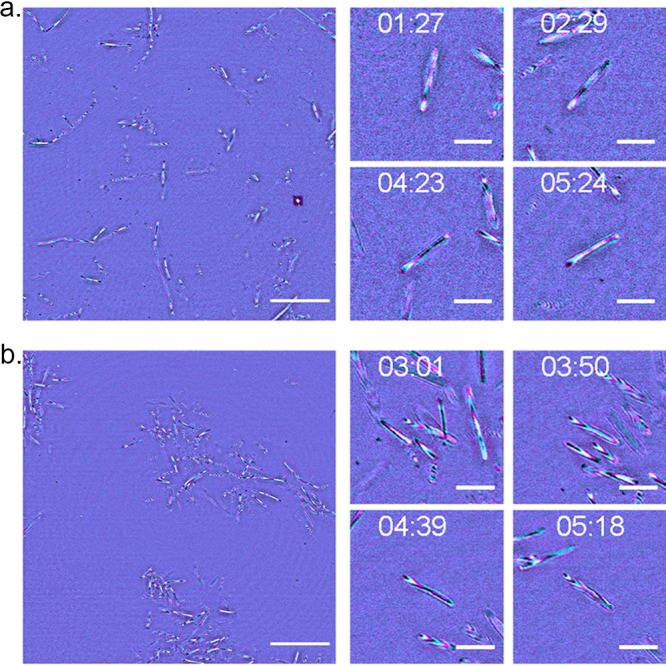
IRM reveals axial movements along the cell body during gliding motility. (a) A single frame from a wild-type DK1622 gliding specimen with 4 magnified regions of interest (ROI) of a single representative cell over the course of the time-lapse (from *t* = 1 min 27 s to *t* = 5 min 24 s). Images were acquired using a multiwavelength approach, with the reflected 488-nm signal false colored in cyan and the reflected 635-nm signal shown in magenta. As the cell glides across the solid substrate, the interference fringe pattern changes as the relative position of the cell to the cover glass fluctuates. (b) A single frame of DK1622 Δ*aglQ* with magnified ROI from a single representative gliding cell over the course of the time-lapse (from *t* = 3 min 1 s to *t* = 5 min 18 s). Images were acquired using incident light at 488 nm (cyan) and 635 nm (magenta). DK1622 Δ*aglQ* exhibits the same axial movements as the wild type, demonstrated by the presence of interference fringes which fluctuate as the cell glides. Full time series data for DK1622 and DK1622 Δ*aglQ* are presented in Movies S1 and S2 in the supplemental material, respectively. Bars = 20 μm (single frame) and 5 μm (ROI).

Using confocal IRM, we observed previously undocumented changes in the axial position of M. xanthus cells as they glide. Figure 3a shows a single frame from a background-corrected (see the supplemental material) multiwavelength confocal IRM time-lapse recording of wild-type cells with a magnified view of a representative cell at different time points throughout the time series (the full data set is provided in Movie S1). In Fig. 3a, clearly resolved interference fringes along the cell body indicate that the cell is not completely attached to the cover glass but that the cell-to-cover glass distance varies along the cell body. There is a clear aperiodic change in the fringe pattern over time, which indicates changes in the adhesion profile of the cell body during gliding. Interpreting the color ordering of these fringes, as with the lens specimen, shows that part of the cell body is lifted up from the glass substrate. This observed change in the adhesion profile opposes the current theory that gliding cells firmly adhere along the cell body as they glide. Height changes also show no synchronicity between nearby gliding cells.

10.1128/mSphere.00846-19.7 MOVIE S1 IRM time series of gliding DK1622 cells. Download Movie S1, AVI file, 8.8 MB. Copyright © 2020 Rooney et al. 2020 Rooney et al. This content is distributed under the terms of the Creative Commons Attribution 4.0 International license.

10.1128/mSphere.00846-19.8 MOVIE S2 IRM time series of gliding DK1622 Δ*aglQ* cells. Download Movie S2, AVI file, 8.4 MB. Copyright © 2020 Rooney et al. 2020 Rooney et al. This content is distributed under the terms of the Creative Commons Attribution 4.0 International license.

Figure 3b shows a single frame from a background-corrected multiwavelength confocal IRM time-lapse recording of a DK1622 Δ*aglQ* strain with a magnified view of a representative gliding cell (the full data set is provided in Movie S2). The deletion of *aglQ* results in a loss of PMF from the AglRQS complex, which in turn prevents the rotation of the MreB cytoskeleton and transit of the FAC along the cell body (23). Figure 3b shows that the fluctuations in cell topology present in the wild type are also present in DK1622 Δ*aglQ*. This conserved motility behavior suggests that axial changes in the cell body during gliding are independent of PMF-driven translocation of FACs along the cell body and are not linked to the proposed membrane protrusions reported in other studies. We then investigated if T4P were responsible for the changes in the adhesion profile that we report. The deletion of the T4P subunit PilA resulted in cells being unable to adhere to the cover glass and initiate gliding (Movies S3 and S4).

10.1128/mSphere.00846-19.9 MOVIE S3 IRM time series of gliding DK1622 Δ*pilA* cells. Download Movie S3, AVI file, 5.0 MB. Copyright © 2020 Rooney et al. 2020 Rooney et al. This content is distributed under the terms of the Creative Commons Attribution 4.0 International license.

10.1128/mSphere.00846-19.10 MOVIE S4 IRM time series of gliding DK1622 Δ*aglQ* Δ*pilA* cells. Download Movie S4, AVI file, 9.4 MB. Copyright © 2020 Rooney et al. 2020 Rooney et al. This content is distributed under the terms of the Creative Commons Attribution 4.0 International license.

### Using multiwavelength IRM for extracting 3D directionality.

**FIG 4:**
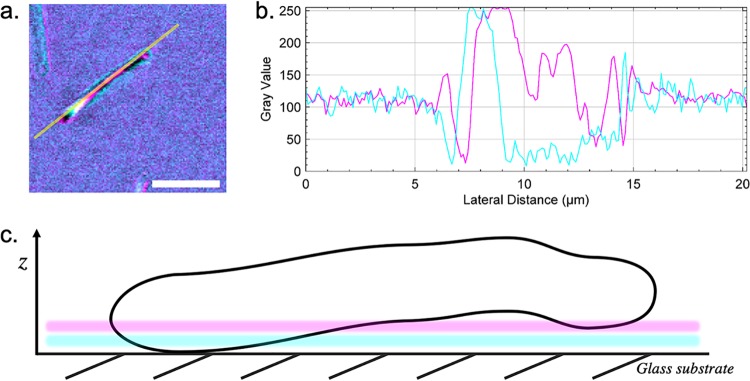
The interference fringe patterns of a gliding cell reveal the axial profile of the cell. (a) A representative DK1622 cell from a time series data set with the location where the intensity profile in panel b was measured. Interference fringes along the cell body can be observed, with the reflected 488-nm signal shown in cyan and the 635-nm signal in magenta. Bar = 5 μm. (b) Intensity plot profile from the line through the cell presented in panel a. The plot shows the maxima and minima of the interference fringes acquired using both 488-nm (cyan) and 635-nm (magenta) light. The spectral separation of the two interference patterns can also be observed. Axial directionality of the cell can be determined by interpreting the color ordering of the fringes, where fringes arising from the longer wavelength appear after those from the shorter wavelength when the cell is inclined, and the opposite is observed for declining slopes. The plot was acquired by averaging the signal over a line width of 3 pixels (0.3 μm). (c) Schematic of the *x*, *z* profile of the cell shown in panel a according to the color ordering and intensity profile in panel b. The axial positions of the colored fringes from each acquisition wavelength are also shown. The cell has not adhered to the solid surface during gliding and does not maintain a linear cylindrical profile along the length of the cell body. According to theory, regions that intersect the axial position of the first-order 488-nm maxima are located 91.7 nm above the substratum, and those for the first-order 635-nm maxima are located 119.3 nm above the substratum. The schematic is not drawn to scale.

Figure 4 illustrates how multiwavelength IRM data can be assessed qualitatively to understand the geometry of a single gliding M. xanthus cell (additional gliding morphologies are presented in Fig. S3). A representative wild-type gliding cell is presented, where multicolor fringes can be observed along the cell body (Fig. 4a). In the IRM image, lifting of the cell body is clearly indicated by an alternating fringe pattern along the body. By interpreting the intensity plot profile along the cell body (Fig. 4b), we can extract qualitative topological information about cell morphology. Figure 4b shows a cyan fringe (λ = 488 nm) followed by a magenta fringe (λ = 635 nm), and based on this, we can conclude that the cell body was attached to the cover glass at the leading pole before the basal surface raised to a height of approximately 180 nm. A cartoon diagram approximating the shape of the cell body is shown in Fig. 4c. The IRM data indicate a variety of cell orientations and shapes that occur during gliding motility. Examples of additional morphologies are presented in Fig. S3. These include an undulating topology where the cell is attached to the cover glass at the leading pole, while the cell body raises to a height of approximately 180 nm before falling to 90 nm and again raising to 180 nm at the lagging pole (Fig. S3a). Another cell motility behavior is depicted at the point of surface attachment prior to the start of gliding, where the leading pole of the cell is attached to the cover glass and the cell body projects upward into the liquid medium at a sharp incline of approximately 60° relative to the cover glass (Fig. S3b).

10.1128/mSphere.00846-19.3 FIG S3 Additional motility behaviors displayed by Myxococcus xanthus. (a) Corrected IRM image of a wild-type gliding cell with an accompanying intensity plot profile (line width = 3 pixels [0.3 μm]). An *x*, *z* schematic is presented, which illustrates an undulating motion of the basal surface of the cell during gliding. (b) Corrected IRM image of a wild-type cell with an accompanying intensity plot profile (line width = 3 pixels [0.3 μm]). The schematic shows the cell attached to the substrate at one pole and projecting upward into the surrounding medium. Reflected 488-nm light is false colored in cyan, and 635-nm light is in magenta. Bar = 5 μm. Download FIG S3, TIF file, 2.8 MB. Copyright © 2020 Rooney et al. 2020 Rooney et al. This content is distributed under the terms of the Creative Commons Attribution 4.0 International license.

### Using IRM to measure the velocity of gliding cells.

**FIG 5:**
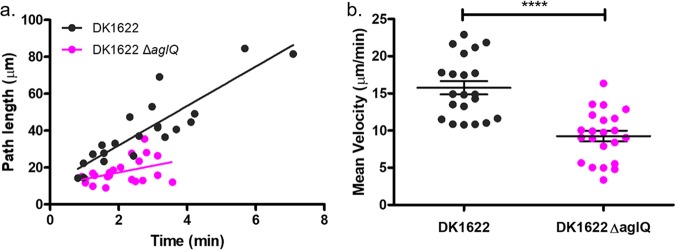
IRM as a method for measuring the velocity of adherent cells. (a) Path lengths of DK1622 and DK1622 Δ*aglQ* over the course of a time series. Overall, DK1622 has an increased mean path length compared to DK1622 Δ*aglQ*, which shows that DK1622 Δ*aglQ* has a lower adhesion profile than the wild type. (b) Deletion of *aglQ* results in a decrease in the mean velocity of 41.2%, from 15.76 ± 0.89 μm/min to 9.26 ± 0.72 μm/min, compared with the wild type (*n*_DK1622_ = 21; *n*_DK1622 Δ_*_aglQ_* = 22 [****, *P* < 0.0001]).

We used IRM to determine the mean velocity of motile cells and showed that the deletion of *aglQ* decreased the length of time that cells remain adhered to the glass substrate compared to the wild type (Fig. 5a). This implies that AglQ is responsible for maintaining adherence to the cover glass during gliding. The mean velocity of gliding cells was determined by measuring the displacement of the cells over time as they glide, selecting cells with an approximately linear trajectory. We found a 41.2% decrease in the mean velocity of DK1622 Δ*aglQ* (mean velocity = 9.26 ± 0.72 μm/min) compared with the wild type (mean velocity = 15.76 ± 0.89 μm/min), which concurs with their altered motility phenotype (Fig. 5b).

## DISCUSSION

We report aperiodic changes in the adhesion profile of gliding myxobacteria using the label-free technique IRM. Given that previous studies had failed to identify any axial behaviors in gliding cells due to the drawbacks of conventional imaging techniques, we hypothesized that changes in cell height from the substrate may arise due to the complex mechanisms that govern gliding motility. The data presented here show new behavior in gliding myxobacteria that do not support the current gliding motility models. The behaviors that we report suggest that there are additional factors that mediate gliding motility and show the benefit of using IRM to extract 3D information from bacterial specimens by using an easily implemented microscopy technique.

The current consensus is that gliding cells must be firmly attached to a solid surface to facilitate gliding; however, these data show that this is not the case. Our results show that throughout gliding, cells undergo changes in the axial position of their basal surface on the order of 90 to 180 nm. This new information indicates that the helical rotation and FAC motility models do not fully explain the mechanisms of bacterial gliding. One previous study used RICM to investigate the adhesion profile of myxobacteria during detachment (17). However, those authors did not report any fringes along the cell body or any aperiodic changes in adhesion in gliding cells. This is likely due to the poor contrast of wide-field IRM and RICM compared to confocal IRM, which prohibits the detection of higher interference fringe orders. It is important to also note that some researchers have claimed that the ability of IRM to detect regions of close contact is questionable due to the inhomogeneous refractive index of the cytosol proximal to the plasma membrane and from self-interference from higher fringe orders within the cell (44, 46). However, these claims are based solely on observations of mammalian cells using wide-field IRM. Owing to the lack of vesicular trafficking and the much smaller relative size of bacteria than mammalian cells, we maintain that confocal IRM remains a valid technique for studying surface adhesion in prokaryotes.

Previous studies that have used conventional optical microscopy methods to image gliding myxobacteria have also failed to observe changes in surface adhesion due to the elongation of the axial point spread function being on the same order as the thickness of a bacterial cell (51, 52). By adopting a confocal approach to IRM for bacterial imaging, we overcome the limits of optical sectioning of thin specimens in standard optical microscopy while simultaneously visualizing the adhesion profile of cells. The drawback of using confocal IRM is a decreased temporal resolution compared with that of wide-field IRM. However, we were still able to track gliding cells sufficiently. While the contrast improvement provided by using a confocal approach over wide-field microscopy should make image analysis easier (52, 53), the complexity of IRM image data makes image processing and analysis challenging. There are currently no readily available image-processing tools that can extract 3D information from a 2D image, such as in IRM. However, with these improved data, it may be possible to develop software tools to reconstruct the 3D topography of bacteria using IRM data. The temporal resolution could be improved by using a spinning-disk confocal microscope for IRM; however, most commercial spinning-disk microscopes may not be suitable to incorporate IRM due to hardware limitations (43). The use of confocal IRM also allows us to confirm that the studied cells are in close proximity to the surface of the cover glass and that the gliding behaviors that we report are not caused by other factors such as inertia or Brownian motion.

Changes in the relative position of the basal cell membrane above the cover glass could perhaps be explained by proposed distortions in the cell wall due to the translocation of FACs along the basal surface of the cell (19) or because of cell surface unevenness (54). However, when we compare the axial movements displayed by the wild type with those of the Δ*aglQ* mutant, we see that this novel gilding behavior remains. The role of AglQ in gliding motility is central to both the helical rotation and FAC models (22, 24, 25, 55), and having both wild-type and DK1622 Δ*aglQ* cells display the same behavior highlights that more understanding of the mechanisms of gliding motility is required. We then investigated the role of T4P in the changes that we observe. Time-lapse images of DK1622 Δ*pilA* and DK1622 Δ*aglQ* Δ*pilA* mutants were acquired to establish if T4P-mediated events altered the adhesion behaviors that we observed, but these cells were not able to adhere to the cover glass and did not display an adherence pattern when imaged using IRM. We were therefore unable to confirm if T4P extension, attachment, and retraction were responsible for the behaviors that we document (see Movies S3 and S4 in the supplemental material).

It could be possible that slime secreted from the gliding cell could be responsible for the behaviors that we observe, where slime with a refractive index so close to that of the culture medium was not detected by IRM. This could perhaps be demonstrated by the use of fine graphite particles, scanning electron microscopy, or atomic force microscopy, which are all effective in revealing the otherwise invisible slime secretions. A recent study by Tchoufag et al. proposed an elastohydrodynamic mechanism for bacterial gliding where a sinusoidal basal shape is adopted when myxobacteria glide upon a soft substrate (56). This was observed using membrane-stained gliding cells on semisolid agar pads with TIRF microscopy and revealed distinct foci where the cell membrane was lifted approximately 100 nm above the substrate surface (outside the TIRF evanescent field). The motility behaviors described here are reminiscent of this undulating basal shape; however, our observations are documented for solid glass substrates and describe a wider variety of 3D behaviors in addition to the sinusoidal oscillatory behavior reported by Tchoufag et al. Moreover, our findings indicate that sinusoidal oscillatory gliding behaviors of M. xanthus may be surface independent.

Finally, we measured the mean velocity of wild-type and DK1622 Δ*aglQ* cells. Using the Δ*aglQ* mutant as a control, where we know from previous work that gliding should be impaired (22), we have shown that height-changing behaviors are independent of the gliding velocity or path length where cells remain associated with the cover glass. To determine the gliding velocity, we measured the average time that cells glided along approximately linear trajectories. Routine automated tracking algorithms generally have difficulties in tracking bacterial specimens due to their reliance on blob detection of spherical objects (57–59). Attempts to isolate and track rod-shaped objects, such as M. xanthus, have proven difficult, and the addition of interference fringes to rod-shaped objects only adds to the complications of automated cell tracking and analysis. We therefore used a manual tracking method to measure the mean velocity of gliding cells. We concluded that the DK1622 Δ*aglQ* mutant moved on average 30% slower than the wild type. Also, the wild type remains adhered to the surface for longer periods of time, yet the dynamic fluctuations in gliding adhesion that we report remain.

This work has provided new insights into the 3D motility of bacteria and identified novel motility behaviors in M. xanthus, which suggests that there are additional mechanisms that do not agree with the current FAC and helical rotation models. We suggest that the fluctuations in height that we observe are mediated by T4P and occur due to recoil following firing of pili from the leading pole. In this work, we attempted to image T4P mutants to confirm this hypothesis (Movies S3 and S4); however, these mutants were unable to attach to the glass substrate and therefore were unable to glide. Given that IRM was unable to reveal T4P-mediated behaviors due to nonattachment of cells to the cover glass, we suggest that this hypothesis could be investigated further by means of a correlative IRM method. Moving forward, it would be interesting to investigate the spatiotemporal dynamics of T4P firing in association with the changing adhesion profile of gliding cells using a correlative TIRF-IRM approach. This would allow the simultaneous imaging of the adhesion profiles of gliding cells and fluorescently tagged T4P proximal to the cover glass. Additionally, the development of an image-processing workflow to extract 3D information from confocal IRM images of myxobacteria would provide users with a method to quantify aperiodic oscillatory behavior. Alternatively, an astigmatic approach to single-molecule localization microscopy could be used to image the basal membrane of cells at nanometer resolution. However, this would require laborious specimen preparation, fixation of cells during gliding, and long acquisition times, which would make observations of such dynamic behaviors impractical compared to the gentler label-free nature of IRM. Furthermore, structured illumination microscopy (SIM), which can provide up to two times the resolution of point-scanning microscopy, could be used to obtain high-resolution 3D images. However, SIM would also require cells to be fluorescently labeled, which could alter the behavior of the specimen, whereas this is of no concern in label-free techniques such as IRM.

## MATERIALS AND METHODS

### Bacterial cell culture.

Myxococcus xanthus cultures (Table 1) were maintained on double Casitone yeast extract (DCYE) medium (20 g/liter casein hydrolysate, 2 g/liter yeast extract, 8 mM MgSO_4_, 10 mM Tris-HCl [with 20 g/liter agar for solid medium]). For imaging, cells were inoculated at high cell densities in liquid DCYE medium and grown for 48 h at 30°C with shaking at 250 rpm. Prior to imaging, an 800-μl sample of the exponentially growing culture was removed from liquid culture and placed in a 35-mm optical-bottom petri dish with a coverslip thickness of 180 μm (catalog no. 80136; ibidi GmbH, Germany) and incubated at 30°C for 20 min to allow cells to adhere.

**TABLE 1:** Bacterial strains used in this study

Strain	Characteristic(s)	Reference or source
DK1622	Wild type	59
DK10410	Δ*pilA*	60
SA5293	Δ*aglQ*	53
SA7705	Δ*pilA* Δ*aglQ*	Lotte Søgaard-Andersen, MPI-Institute for Terrestrial Microbiology, Germany

The refractive index of liquid DCYE medium was measured to be 1.33 using an Abbe refractometer (Billingham & Stanley Ltd., UK).

### Interference reflection microscopy.

For the characterization of a model lens specimen, the specimen was placed convex side down on a 170-μm-thick cover glass measuring 50 by 24 mm, which bridged the microscope stage insert. An Olympus IX81 inverted microscope coupled to a FluoView FV1000 confocal scanning unit (Olympus, Japan) was used to image the lens specimen. The microscope was configured for IRM by replacing the emission dichroic with an 80/20 beam splitter. Images were acquired using a 10×/0.3-NA UPlanFl lens objective (Olympus, Japan), and reflection signals were detected using a photomultiplier tube (PMT) for each wavelength, with spectral detection limited to a 10-nm bandwidth over the wavelength of the incident light. A 488-nm line from an argon laser source (model no. GLG3135; Showa Optronics, Japan) was used for single-wavelength acquisition. For multiwavelength acquisition, 488-nm and 514-nm lines were provided by an argon laser and a 543-nm line was provided by a helium-neon-green laser source (model no. GLG3135; Showa Optronics, Japan).

For M. xanthus imaging, optical-bottom dishes were placed on the stage of an inverted Olympus IX81 microscope coupled to a FluoView FV1000 confocal laser scanning unit (Olympus, Japan). Images were acquired using a 60×/1.35-NA UPlanSApo oil lens objective (Olympus, Japan). The microscope was configured for IRM as described above, with incident light of 488 nm from an argon laser and 635 nm obtained from a 635-nm laser diode (model no. GLG3135; Showa Optronics, Japan) for multiwavelength acquisitions. These wavelengths were selected based on their large spectral separation, meaning that color ordering in the observed IRM images was more distinct. For multiwavelength images, both channels were acquired simultaneously with two separate photomultiplier tube detectors.

For wide-field IRM, specimens were prepared as described above and imaged using a Nikon Eclipse-Ti2 inverted microscope (Nikon Instruments, USA) coupled to a Prime 95B sCMOS camera (Teledyne Photometrics, USA). Images were acquired using a 60×/1.4-NA PlanApo oil objective lens (Nikon Instruments, USA). The microscope was configured for IRM by placing an 80/20 beam splitter into the detection path, and incident light was sourced from 450-nm and 550-nm light-emitting diodes (LEDs) (CoolLED, UK). Multiwavelength images were acquired sequentially.

### Image processing and analysis.

**(i) Image correction.** A common problem for the analysis of IRM images is the inhomogeneity of brightness across the image, which limits the utility of image segmentation tools like thresholding. To correct for changes in image brightness across the field, the moving average of the *k* × *k* neighborhood was divided from each pixel using MATLAB 2018b. To rescale the histogram for downstream analysis in Fiji (60), the image intensity was rescaled by dividing by a factor of 2.

The length of the neighborhood, *k*, was selected depending on the specimen that was imaged. For the lens specimen, a large *k* value (*k *= 1,000) was chosen to prevent lowering the contrast of the lens signal. For IRM images of M. xanthus, where the frequency of the observed interference fringes relative to the pixel density is high, a relatively small *k* value (*k *= 30) proved suitable. Line intensity profiles from raw and corrected IRM images were checked to verify that the position and intensity succession of the interference fringes were not altered due to the correction method (see Fig. S4 to S6 in the supplemental material).

10.1128/mSphere.00846-19.4 FIG S4 Image correction workflow for IRM data. (a) Linearly contrast-adjusted IRM image of a 6-mm-diameter lens specimen (focal length = 72 mm) acquired using light at 488 nm. Bar = 100 μm. (b) Linearly contrast-adjusted IRM image of M. xanthus with inhomogeneous background intensity. Bar = 10 μm. (c) Calculated moving average from panel a when *k* is equal to 1,000. The calculated moving average accounts for the inhomogeneous background, which is subtracted to correct the raw image. (d) Calculated moving average from panel b when *k* is equal to 30. (e) Corrected IRM image resulting from panel a. (f) Corrected IRM image resulting from panel b. The ROI selects a single cell where the intensity profile was measured in this figure. Selected line plots are shown in each panel where intensity profiles were measured in Fig. S3 in the supplemental material. Download FIG S4, TIF file, 2.2 MB. Copyright © 2020 Rooney et al. 2020 Rooney et al. This content is distributed under the terms of the Creative Commons Attribution 4.0 International license.

**(ii) Lens analysis and reconstruction.** In this work, we verified that multiwavelength IRM can be used to study the change in cell topology during gliding by imaging a lens specimen of known geometry and comparing the results with the theoretical model (see “Background theory,” above). The one-dimensional height profile of a lens, *z*_lens_, is $z(x)=R-\sqrt{R^{2}-x^{2}}$, where *R* is the radius of the lens and *x* is the distance to the center point of the lens that touches the surface/coverslip (*x *= 0; *z *= 0).

Images acquired at multiple wavelengths of the lens specimen were linearly contrast adjusted and cropped to create a square image using Fiji (60). A composite red/green/blue (RGB) image was created by merging the channels. The data were imported into MATLAB 2018b, and using the same radial analysis method as the one presented by Tinning et al., we calculated the axial height of interference fringes from the composite RGB image of the lens specimen (61). We used a findpeaks function to determine the spacing between the experimental intensity maxima of the interference fringes. Each subsequent constructive interference fringe was subtracted from its neighboring fringe to calculate the experimental spacing.

Lens reconstruction was performed using MATLAB. First, the radial distance for each pixel was extracted from the center of the RGB IRM image of the lens specimen. By applying *a priori* knowledge of the lens specimen geometry to the radial distance of each pixel (see “Background theory,” above), we were able to assign each pixel to an axial height value based on the experimental fringe separation calculated previously. The *x*, *y*, and *z* coordinates along with the image intensities were plotted to create a 3D reconstruction of the RGB IRM image.

10.1128/mSphere.00846-19.5 FIG S5 Image correction does not alter the position of fringe maxima and minima. (a) Selected line intensity profiles from Fig. S4a, c, and e in the supplemental material. Following correction of the raw images using the calculated moving average, there was no change in the peak position of the intensity maxima and minima. (b) Selected line intensity profiles from Fig. S4b, d, and f. Correction resulted in homogeneity of illumination across the field. Line intensity profiles were averaged using line widths of 5 pixels (4.2 μm) (a) and 5 pixels (0.5 μm) (b). Download FIG S5, TIF file, 1.8 MB. Copyright © 2020 Rooney et al. 2020 Rooney et al. This content is distributed under the terms of the Creative Commons Attribution 4.0 International license.

10.1128/mSphere.00846-19.6 FIG S6 Image correction does not alter the position of intensity peaks. (a) Unprocessed ROI from Fig. S4f in the supplemental material with a line profile shown for reference. (b) Corrected ROI shown in panel a (*k *= 30). (c) Selected line intensity profiles from panels a and b showing that there are no changes in the position of intensity values along the length of gliding cells. Line intensity profiles were averaged using a line width of 5 pixels (0.5 μm). Download FIG S6, TIF file, 2.9 MB. Copyright © 2020 Rooney et al. 2020 Rooney et al. This content is distributed under the terms of the Creative Commons Attribution 4.0 International license.
