# Supplementary figures and images for: Three-Dimensional Observations of an Aperiodic Oscillatory Gliding Behavior in Myxococcus xanthus Using Confocal Interference Reflection Microscopy
Source: mSphere. 2020 Jan 29;5(1):e00846-19. doi: 10.1128/mSphere.00846-19 (PMC6992375; doi:10.1128/mSphere.00846-19)

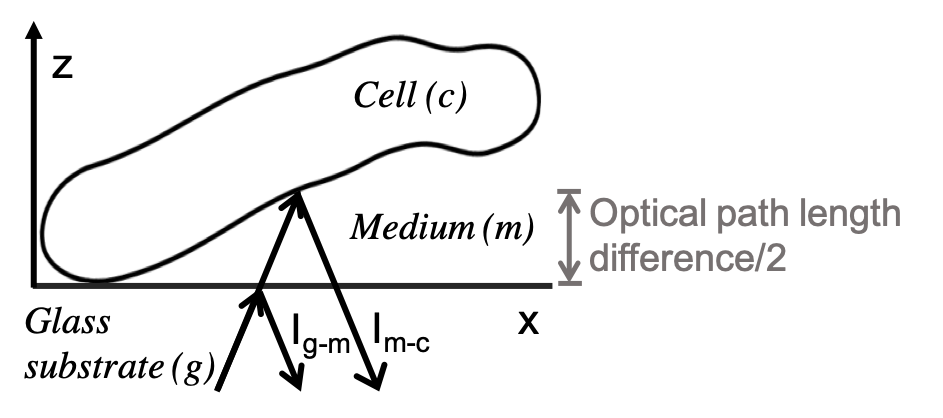

Supplement: FIG S1 [file mSphere.00846-19-sf001.tif]

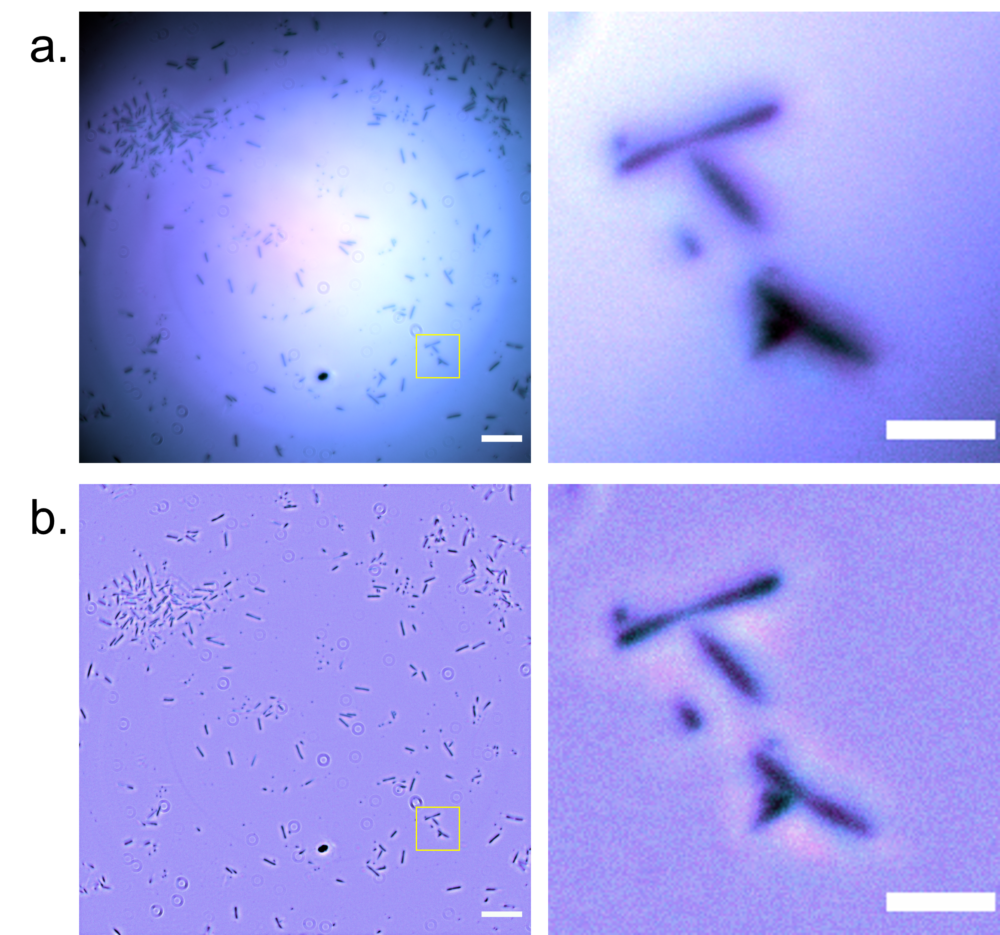

Supplement: FIG S2 [file mSphere.00846-19-sf002.tif]

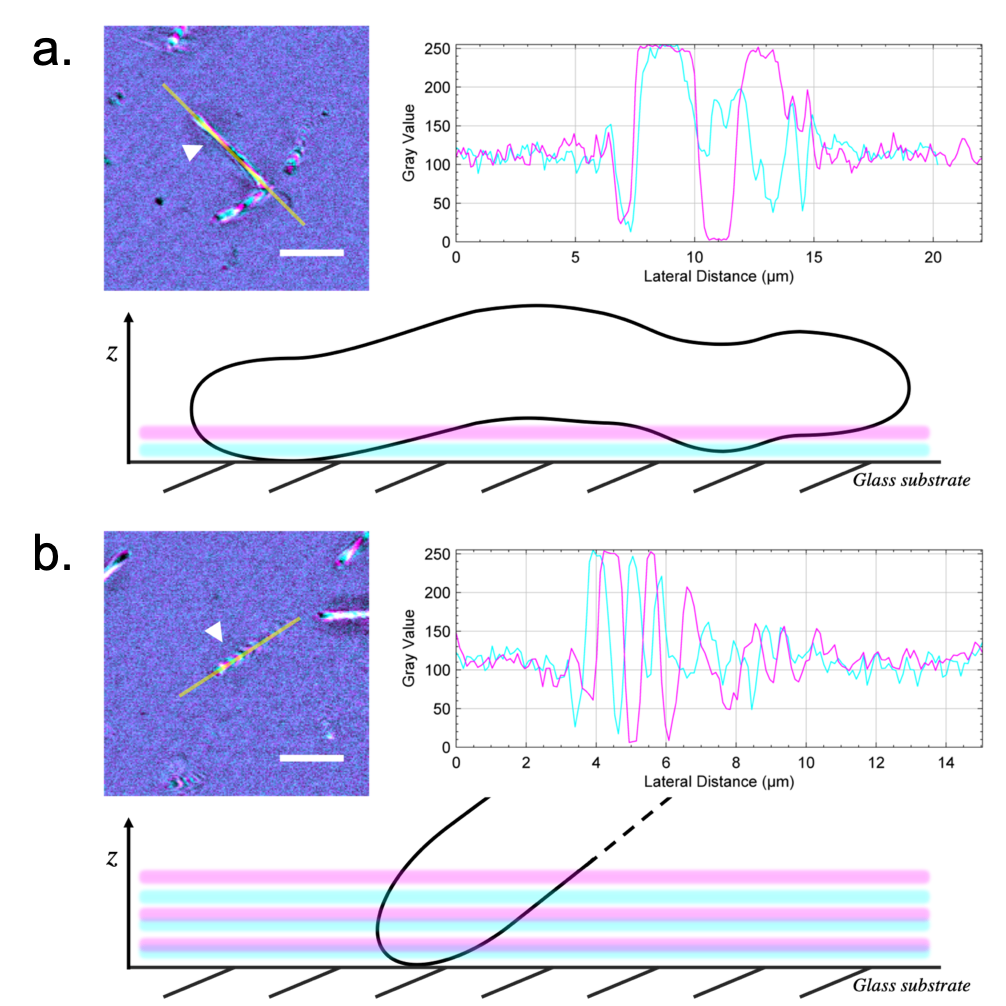

Supplement: FIG S3 [file mSphere.00846-19-sf003.tif]

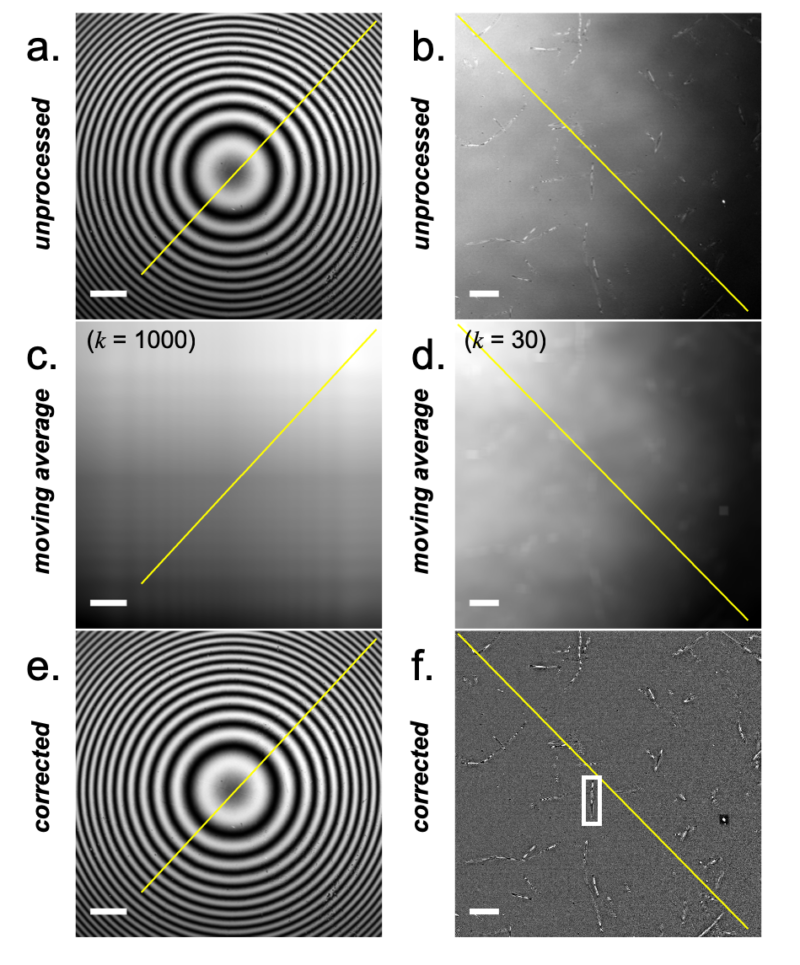

Supplement: FIG S4 [file mSphere.00846-19-sf004.tif]

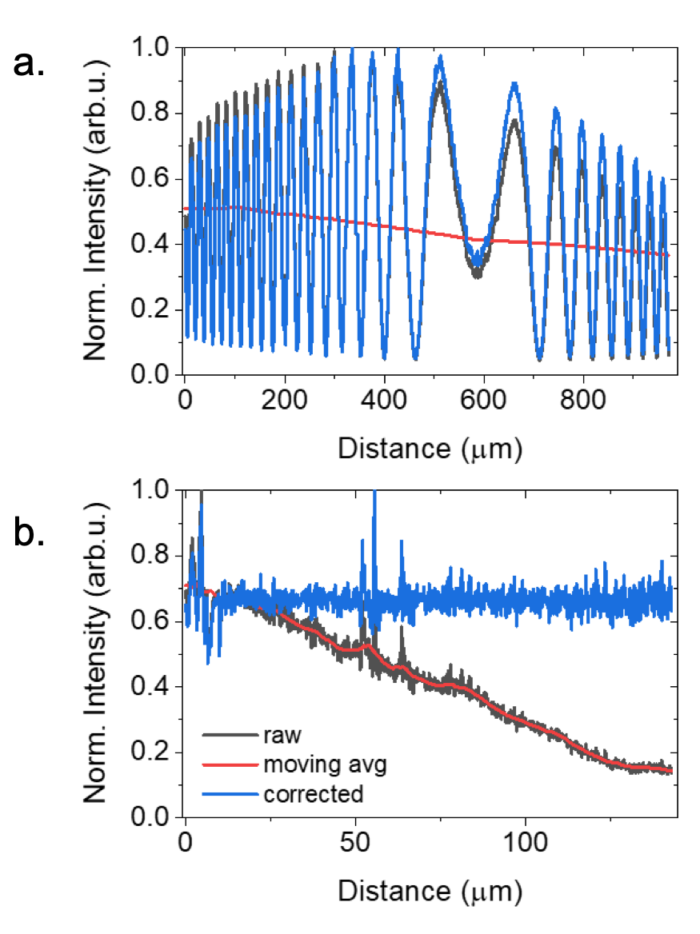

Supplement: FIG S5 [file mSphere.00846-19-sf005.tif]

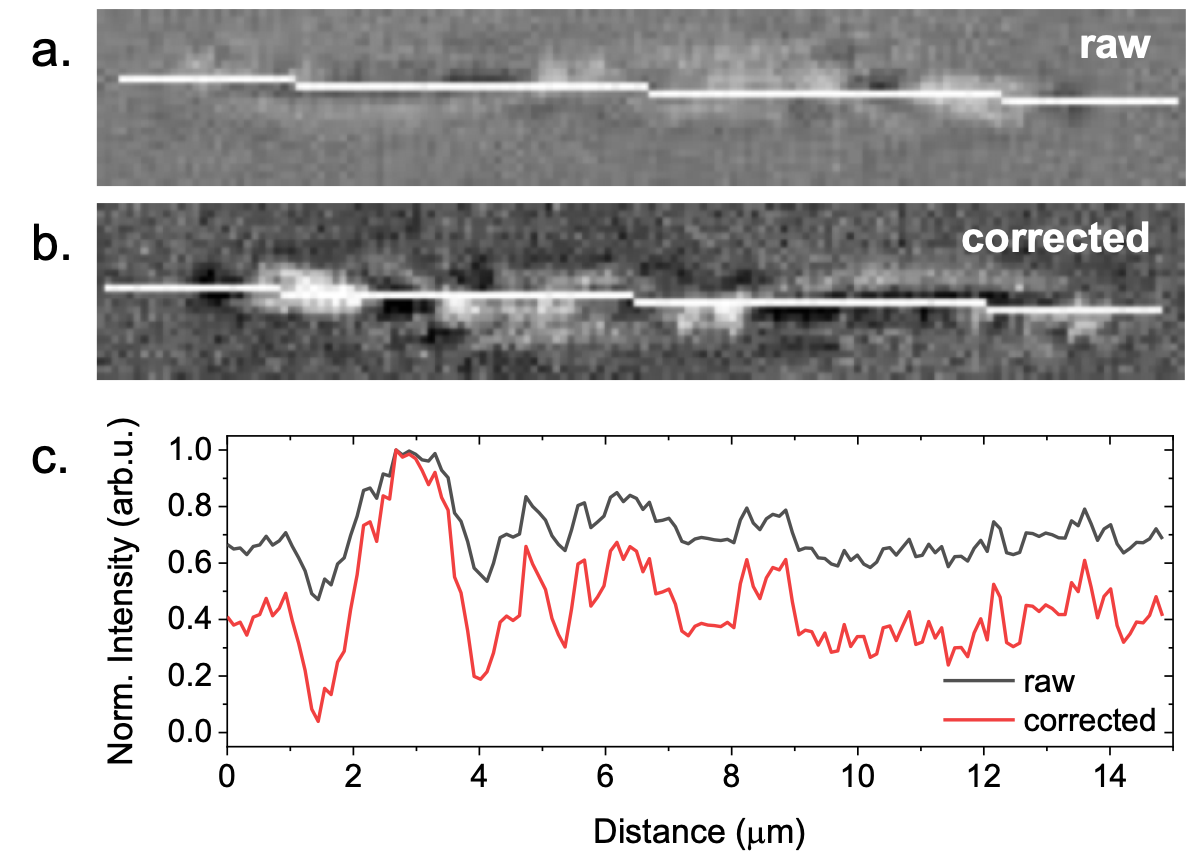

Supplement: FIG S6 [file mSphere.00846-19-sf006.tif]
